# Relation between CarS expression and activation of carotenogenesis by stress in *Fusarium fujikuroi*

**DOI:** 10.3389/fbioe.2022.1000129

**Published:** 2022-10-05

**Authors:** Macarena Ruger-Herreros, Steffen Nordzieke, Carmen Vega-Álvarez, Javier Avalos, M. Carmen Limón

**Affiliations:** Department of Genetics, Faculty of Biology, University of Seville, Seville, Spain

**Keywords:** light, CarS, photoinduction, oxidative stress, heat shock, nitrogen starvation, intron, alternative splicing

## Abstract

*Fusarium fujikuroi,* a model organism for secondary metabolism in fungi, produces carotenoids, terpenoid pigments with antioxidant activity. Previous results indicate that carotenoid synthesis in *F. fujikuroi* is stimulated by light or by different stress conditions and downregulated by a RING finger protein encoded by *carS* gene. Here, we have analyzed the effects of three stressors, nitrogen scarcity, heat shock, and oxidative stress. We compared them with the effect of light in the wild type, a *carS* mutant that overproduces carotenoids, and its complemented strain. The assayed stressors increase the synthesis of carotenoids in the three strains, but mRNA levels of structural genes of carotenogenesis, *carRA* and *carB*, are only enhanced in the presence of a functional *carS* gene. In the wild-type strain, the four conditions affect in different manners the mRNA levels of *carS*: greater in the presence of light, without significant changes in nitrogen starvation, and with patent decreases after heat shock or oxidative stress, suggesting different activation mechanisms. The spores of the *carS* mutant are more resistant to H_2_O_2_ than those of the wild type; however, the mutant shows a greater H_2_O_2_ sensitivity at the growth level, which may be due to the participation of CarS in the regulation of genes with catalase domains, formerly described. A possible mechanism of regulation by heat stress has been found in the alternative splicing of the intron of the *carS* gene, located close to its 3′ end, giving rise to the formation of a shorter protein. This action could explain the inducing effect of the heat shock, but not of the other inducing conditions, which may involve other mechanisms of action on the CarS regulator, either transcriptionally or post-transcriptionally.

## Introduction

**FIGURE 1:**
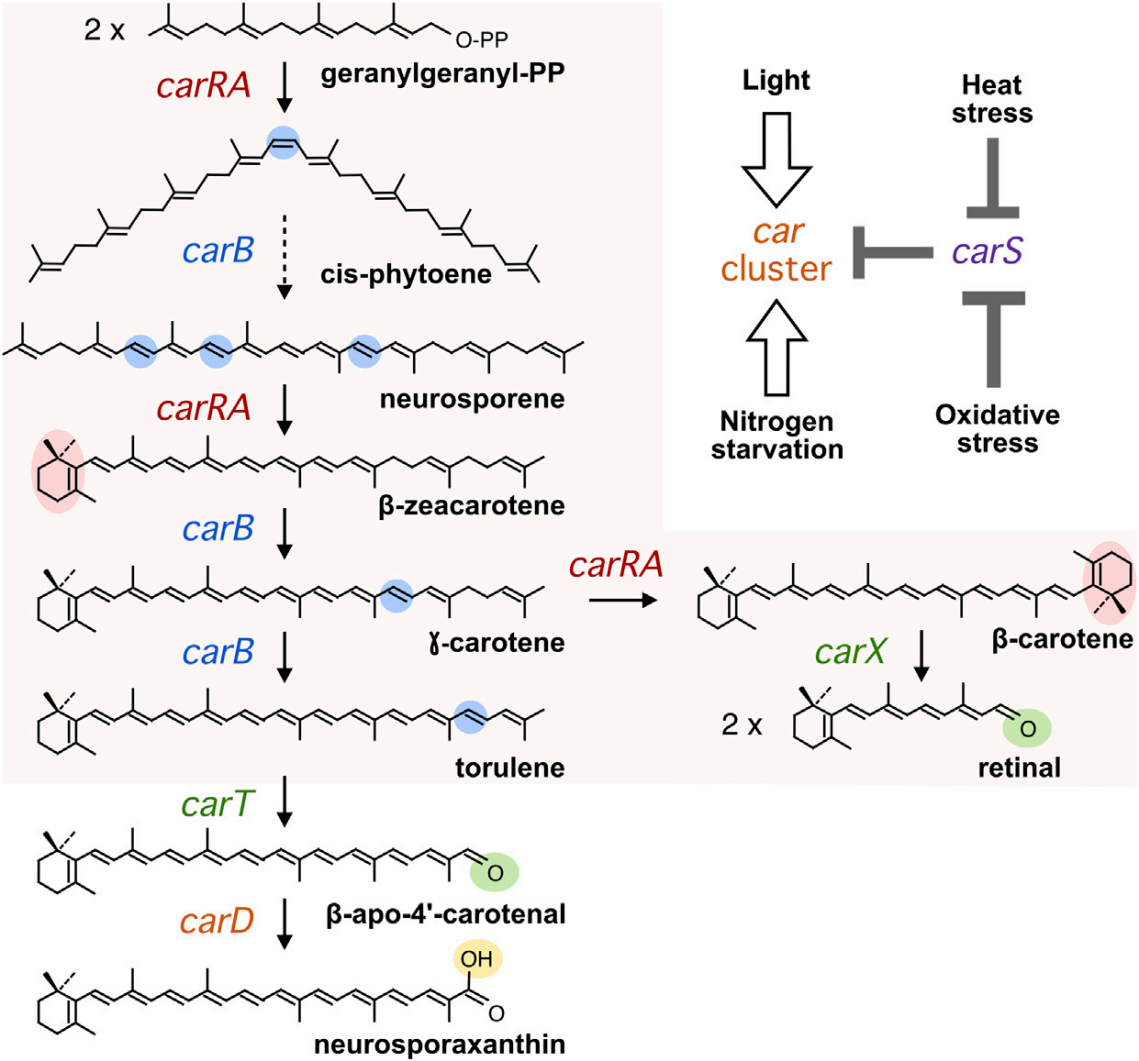
Carotenoid biosynthesis in *Fusarium*. Chemical modifications in each step are highlighted in color. The dashed arrow summarizes three sequential desaturations. The *car* genes encoding the enzymes in charge of each reaction are indicated. The orange area indicates the steps in charge of the genes of the *car* cluster. In the top on the right, it is depicted a simplified model for the effects of the four inducing conditions mentioned in the text (White arrows indicate positive effects on transcription of the *car* genes and grey bars indicate negative effects on *carS* transcription).

The rice pathogen *Fusarium fujikuroi* is an ascomycete fungus well known for its capacity to produce gibberellins (Tudzynski, 2005), plant growth-promoting hormones with applications in agriculture. This species is also a model to produce other compounds, including bikaverin, fusarins, and carotenoids. The carotenoid biosynthetic pathway has received much attention in recent years in this fungus (Avalos et al., 2017), and all genes responsible for the production of its major carotenoid product, the acidic xanthophyll neurosporaxanthin (NX), have been elucidated. Two major genes in the pathway, *carRA* and *carB*, encode a bifunctional phytoene synthase and a desaturase, respectively, and are needed for the synthesis of *β*-carotene and the NX precursor torulene (Figure 1). The *carRA* and *carB* genes are linked to the *carX* gene, encoding a *β*-carotene-cleaving oxygenase (Prado-Cabrero et al., 2007b), and *carO* gene, encoding a proton-pumping rhodopsin (García-Martínez et al., 2015), forming a co-regulated cluster. The CarX oxygenase produces retinal, the rhodopsin chromophore, that gives sense to the coregulation of the cluster as a gene set involved in the production of photoactive CarO rhodopsin. Two other genes, *carT* and *carD,* are unlinked to this cluster and are specifically involved in NX production: CarT as a torulene-cleaving dioxygenase (Prado-Cabrero et al., 2007a) and CarD as an aldehyde dehydrogenase responsible for the formation of the carboxylic group of NX (Díaz-Sánchez et al., 2011).

The synthesis of carotenoids in *Fusarium* is modulated by environmental cues, with light as a major stimulatory signal (Avalos and Estrada, 2010). Illumination of dark-grown mycelia of *F. fujikuroi* results in the acquisition of an orange pigmentation due to an accumulation of carotenoids. This photoresponse is achieved at transcription level, with a rapid but transient increase in the mRNA amounts of the genes of the *car* cluster following illumination, reaching maximal levels at about 1 hour after illumination (Prado et al., 2004). At least three photoreceptors, the White-Collar protein WcoA, the DASH cryptochrome CryD, and the VIVID-like protein VvdA, participate in the control by light of carotenogenesis in *F. fujikuroi* (Castrillo and Avalos, 2015). The mutants of the genes for these photoreceptors exhibit different regulatory alterations, but still retain the ability to accumulate carotenoids in response to light.

Carotenogenesis in *F. fujikuroi* is down-regulated by a RING-finger protein, called CarS. This is evidenced by the outstanding deep-pigmented phenotype in the dark of different mutants of the *carS* gene, and the recovery of the albino appearance by reintroduction of the wild-type *carS* allele (Rodríguez-Ortiz et al., 2013). A similar phenotype is exhibited by equivalent mutants in *Fusarium oxysporum*, where this gene was initially identified (Rodríguez-Ortiz et al., 2012). The increased carotenoid content of the *carS* mutants is due to the enhanced mRNA levels of the structural genes of the pathway either in the dark or under illumination (Prado et al., 2004; Thewes et al., 2005; Prado-Cabrero et al., 2007a; Díaz-Sánchez et al., 2011). The *carS* mutants are reminiscent of *crgA* gene mutants of *Mucor circinelloides* with a deep-pigmented phenotype, due to overexpression of the *car* genes and overaccumulation of *β*-carotene (Navarro et al., 2000). CarS and CrgA are orthologous proteins, containing two RING finger domains and a LON protease domain. Despite the high phylogenetic divergence between both fungi, belonging to the Ascomycotina and Mucormycotina taxa, the coincidences in protein domains and mutant phenotypes suggest similar mechanisms of action. The data available on CrgA points to an ubiquitinating protein that can modulate the function of specific protein targets (Silva et al., 2008).

The connection between CarS and the regulations by light has been recently analyzed at the global transcriptome level. RNA-seq analyses carried out on the wild type, a *carS* mutant, and a *carS-*complemented strain, grown in the dark or exposed to light for 1 hour, revealed that light and CarS alter the expression of a large battery of genes (Ruger-Herreros et al., 2019). Interestingly, it was found an outstanding overlap between the genes induced by light and by the *carS* mutation, including those of the *car* cluster, suggesting regulatory connections between CarS and the light-regulating machinery.

The regulation of carotenogenesis in *Fusarium* is also connected to different stress conditions. Thus, nitrogen starvation has been described as a stimulatory factor, as inferred from the increased carotenoid content when the fungus was grown under nitrogen-limiting conditions or when a non-stressed culture was transferred to a nitrogen-free solution (Rodríguez-Ortiz et al., 2009). Moreover, experimental evidence from different fungi points to a protective role of carotenoids against oxidative stress (Avalos and Limón, 2015). In *Fusarium aquaeductuum*, carotenoid content increases in mycelia exposed under red light to methylene blue or toluidine blue which produce singlet molecular oxygen (^1^O_2_) at this wavelength (Lang-Feulner and Rau, 1975). In addition, exposure to hydrogen peroxide (H_2_O_2_) increases carotenoid accumulation in different fungi (Avalos and Limón, 2015), including *F. aquaeductuum* (Bindl et al., 1970). The regulation by light may be related to the association between illumination and some stressing conditions, such as exposure to air (oxidative stress), desiccation (osmotic stress), and high temperature (heat stress). In the latter case, an increase in mRNA levels has been described for the *carB* gene after exposure of *F. fujikuroi* to 42°C during 1 or 2 hours (Prado et al., 2004). Noteworthy, global transcriptomic data revealed that among the genes more affected by light or by the *carS* mutation there are several genes putatively involved in stress (Ruger-Herreros et al., 2019).

To increase our knowledge on the regulatory connection between carotenoid biosynthesis and environmental stress in *F. fujikuroi* and the possible participation of CarS in such relation, we investigated under equivalent culture conditions the effects of light, nitrogen starvation, heat shock, and hydrogen peroxide on carotenoid accumulation. To do this, we employed the wild type, a *carS* mutant, and its complemented strain formerly used in our global transcriptomic studies (Ruger-Herreros et al., 2019). The data were completed with the effect on expression of the key structural *carRA* and *carB* genes, as well as that of the *carS* gene*.* We found similar effects on carotenoid accumulation and *carRA*/*carB* expression, but different effects on *carS* gene expression, suggesting that the CarS protein is not involved in the same manner in the four responses. Special attention has been paid to the effect of the *carS* mutation on hydrogen peroxide sensitivity. Finally, we found alternative splicing events in an intron in the 3′ region of the mRNA of the *carS* gene and verified its possible involvement in stress regulation. The results are consistent with a possible participation in the activation of carotenogenesis by heat shock, but not by other stressors.

## Materials and methods

### Strains and culture conditions

The *Fusarium fujikuroi* strains were the wild type IMI58289, obtained from the Imperial Mycological Institute (Kew, Surrey, England), the SG39 *carS* mutant (Avalos and Cerdá-Olmedo, 1987), and the SG256 complemented strain, isolated from a sectoring complementing strain obtained by introduction of the wild-type *carS* allele in the SG39 mutant (Rodríguez-Ortiz et al., 2013; Ruger-Herreros et al., 2019).

The strains were cultured in DG minimal medium, containing per liter 30 g glucose, 3 g NaNO_3_, 1 g KH_2_PO_4_, 0.5 g KCl, 0.5 g MgSO_4_·7H_2_O, and 2 ml of a microelement solution (Avalos et al., 1985). To obtain spores, the *F. fujikuroi* strains were grown for 8–10 days under illumination at 22°C on EG medium, containing 1 g glucose, 1 g yeast extract, 1 g NO_3_NH_4_, 1 g KH_2_PO_4_, 0.5 g MgSO_4_·7H_2_O, and 16 g agar per liter. When indicated, mycelia were illuminated by exposure to a set of four fluorescent tubes (Philips TL-D 18W/840) at *ca.* 60 cm, providing a light intensity of 7 W m^-2^.

For RT-PCR analyses, 10^6^ spores of each strain were inoculated into 100 ml of DG medium in 500 ml Erlenmeyer flasks and incubated in the dark for 3 days in an orbital shaker at 200 rpm at 30°C. Afterwards, 25-ml samples of the cultures were transferred to 8.9-cm diameter Petri dishes under red safelight and incubated without agitation for 1 hour in the dark, under white light or subjected to heat shock or to hydrogen peroxide. In the case of nitrogen starvation, incubations in DG medium in 500 ml Erlenmeyer flasks and subsequent transfers to Petri dishes were done with either 3 g L^−1^ NaNO_3_ as high nitrogen condition or with 0.625 g L^−1^ NaNO_3_ as low nitrogen condition. Mycelial samples were collected by filtration and frozen immediately at –80°C for future use. For analysis of carotenoid production, the Petri dishes with the 25 ml of cultures of each strain were incubated for 24 h under the indicated conditions. In all the cases, control plates were incubated in the dark or without stress. For studying the effect of temperature on intron splicing, the Petri dishes with the 25 ml samples were incubated for 1 hour at 16, 22, 30, 37 or 42°C in the dark to adapt to the new temperature. One of the dishes was incubated under illumination and the other in darkness for another hour at the indicated temperature.

### Carotenoid analysis

Mycelial samples were filtered from liquid cultures and frozen in liquid nitrogen for lyophilization. Carotenoids from lyophilized mycelia were extracted with acetone following a standard protocol (Marente et al., 2020b), with two pulses of 6 m/s for 30 s in a FAST- PREP24 (MP Biomedicals, Irvin, CA, United States). Extracted carotenoids were concentrated and measured in a UV-1800 UV-VIS spectrophotometer (Shimadzu, Kyoto, Japan) as previously described (Marente et al., 2020b).

### RNA isolation

Frozen samples of 2–3 mg of mycelium were ground with a FAST-PREP24 (Biomedicals, LLC Europe, France) using zirconia/silica beds with two pulses of 30 s at 4 m/s. Total RNA was extracted using RNeasy^®^ Plant Mini Kit (QIAGEN GmbH, Germany) following manufacturer instructions and specific indications for fungal samples.

### Retrotranscription

Before retrotranscription, DNA contaminations were removed from total RNA samples by treatment with 2 units of rDNAse I RNAse-free (Affymetrix, Santa Clara, CA, United States) per 1 µg RNA for 15 min at room temperature. After inactivation at 65°C, RNA concentrations were estimated with a Nanodrop ND-1000 spectrophotometer (Nanodrop Technologies, Wilmington, DE, United States). 2.5 μg of RNA were retrotranscribed to cDNA with the Transcriptor first-strand cDNA synthesis kit (Roche, Mannheim, Germany), following manufacturer instructions.

### Expression analysis

Transcript levels of the investigated genes were analyzed by quantitative real time PCR (qRT-PCR). Final concentrations of cDNA were set to 25 ng μl^−1^. RT-qPCR analyses were performed in a LightCycler 480 real-time instrument (Roche) with the LightCycler 480 SYBR green I Master (Roche), using the primers included in Table 1. Transcript levels for each gene were normalized against the β1-tubulin *FFUJ_04397* gene mRNA levels under the same conditions.

**TABLE 1:** Oligonucleotides used in this study.

Oligonucleotide	Sequence (5’->3′)
qRT-PCR analyses	
RTcarRA-1F	CAG​AAG​CTG​TTC​CCG​AAG​ACA
RTcarRA-1R	TGC​GAT​GCC​CAT​TTC​TTG​A
RTcarB-1F	TCG​GTG​TCG​AGT​ACC​GTC​TCT
RTcarB-1R	TGCCTTGCCGGTTGCTT
RTFfcarS-1F	GAT​ACC​CGG​CGG​AAA​GGT​TA
RTFfcarS-1R	CTG​ACA​GTC​CAT​TTC​AGC​GC
Tub-2F	CCG​GTG​CTG​GAA​ACA​ACT​G
Tub-2R	CGA​GGA​CCT​GGT​CGA​CAA​GT
Detection of intron splicing by PCR	
carS-11F	TGT​TGA​CTA​TAT​ACG​GCG​AG
S1-carS-1F	GCC​AAC​TTT​CAT​CAG​CTA​GG
carS-11R	CTA​CCG​TCG​AGT​CGT​AAT​CGT​C
carB2intr1F	TGCTGGTGTCGGTGGTGT
carB2intr1R	CGCCCTCAGCAGTTAGAG

### Intron analysis

Alternative intron splicing was identified from junctions between exons in the single readings of RNA-seq analyses of the wild type in a former work (Ruger-Herreros et al., 2019), using Integrative Genomics Viewer application (IGV) version 2.13.1 (Robinson et al., 2011).

Alternative splicing was checked by PCR using 50 ng of cDNA obtained from mRNA extracted from mycelia grown under different conditions. Standard PCR reactions were done in a TC-312 thermal cycler (Techne, Cole Parmer, Vernon Hills, IL, United States), using 50 mM Biotaq polymerase (Bioline, Meridian Bioscience, Cicinnati, OH, United States), 10 mM dNPTs, and 500 mM of primers that are described in Table 1. For *carS* gene, PCR program consisted in 94°C 2 min, 32 cycles of 94°C 30 s, 54°C 30 s, 72°C 30 s, and an extension at 72°C for 10 min. For amplification of longer bands with S1-carS-1F and carS-11R primers, annealing was done at 58°C for 1 min. For *carB* gene, primer annealing was done at 53°C. PCR products were visualized in 1.2% or 2.5% agarose gels depending on their sizes.

To check the validity of the number of cycles, two samples with differences in the patterns of bands were amplified using from 20 to 35 cycles. As a result, amplification signals began to be detected from 26 cycles. Despite increasing the intensity of the bands, the proportions between the bands corresponding to different splicing variants were maintained up to the maximum number of 35 cycles used (Supplementary Figure S1). The band pattern was especially visible at 32 cycles, so these analysis conditions were used in subsequent experiments.

Densitometry analysis was done online with the ImageJ software through the server https://ij.imjoy.io/.

### Germination assay

Spore suspensions of each strain were prepared with approximately 10^7^ spores in 1-ml of water. H_2_O_2_ was added to a final concentration ranging from 25 to 150 mM and controls without H_2_O_2_ were included. The spores were incubated in agitation at 30°C in darkness and after the treatment they were washed twice with sterile water by centrifugating at 3000 × *g* for 5 min, at 4°C. Different dilutions of the suspensions were streaked over DG agar medium. The number of the colonies on each plate was counted after 3 days of incubation at 30°C. Germination of spores was calculated as a percentage of the number of colonies appearing after incubation spores with H_2_O_2_ respect to the non-treated ones.

## Results

### Relation of light and nitrogen starvation with *carS* expression

**FIGURE 2:**
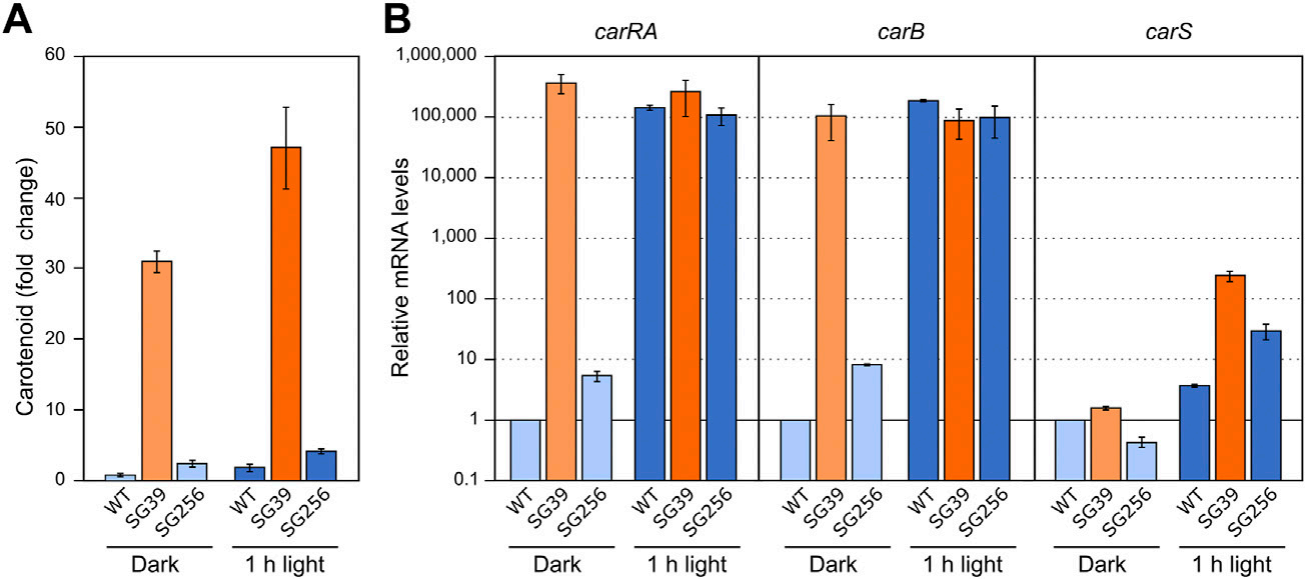
Effect of light and *carS* gene mutation on carotenoid production and expression of *car* genes. **(A)** Relative carotenoid content in the wild type (WT), *carS* mutant (SG39), and the complemented mutant (SG256). Absolute carotenoid contents are shown in Supplementary Table S1. Carotenoids were analyzed in mycelia incubated for 24 h after the 1-h illumination in the Petri dishes or without illumination. **(B)** Transcript levels of *carRA, carB,* and *carS* genes. The three strains were cultivated for 3 days in shake flasks with DG medium at 30°C in darkness, 25 ml of the cultures were transferred to Petri dishes and were illuminated with white light for 1 hour or incubated in darkness. Levels of mRNA were determined by RT-qPCR and referred to the levels in the wild type grown in the dark, taken as 1.

Light and nitrogen starvation have been described as stimulatory signals for carotenogenesis. To assess its possible connection with *carS* regulation, the effect of these signals was checked in the wild type, the *carS* mutant SG39, and the complemented strain SG256. There was a small increase in the carotenoid content of the three strains after exposition to light, while the *carS* mutant exhibited an approximately ten-fold increase compared to *carS*+ controls, both in the dark and after illumination (Figure 2A; absolute carotenoid content for all figures with carotenoid data shown in Supplementary Table S1).

RT-qPCR assays showed that both *carRA* and *carB* transcripts strongly increased in the wild type and in the complemented strain 1 hour after the light exposure, while they were very high in the *carS* mutant irrespective of illumination (Figure 2B). The transcript levels of the *carS* gene were not affected by the *carS* mutation in the dark. As formerly reported, a modest photoinduction of such levels was observed in the wild type, but the increase was more pronounced in the *carS* mutant, suggesting a down-regulation of *carS* photoinduction by its own CarS protein. The photoinduction of the *carS* gene in the *carS* mutant contrasted with the lack of effect of light on the mRNA levels for the *carRA* and *carB* genes in the same strain.

Formerly, the effect of nitrogen on carotenogenesis was investigated under different culture conditions (Rodríguez-Ortiz et al., 2009). For better comparison with the effect of light, growth conditions were as described above, but in this case the mycelia were grown for 3 days in minimal medium with high and low nitrogen concentration from the start of the experiment. The N-limiting concentration was inferred from the effect of nitrogen availability on the synthesis of gibberellins (Candau et al., 1992).

**FIGURE 3:**
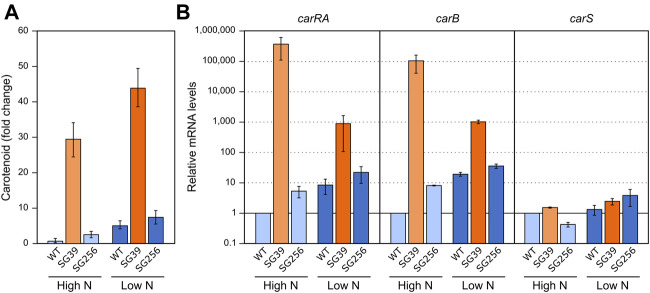
Effect of nitrogen availability and *carS* mutation on carotenoid production and expression of *car* genes. **(A)** Relative carotenoid production or without treatment (control) in the wild type (WT), *carS* mutant (SG39), and the complemented mutant (SG256) under two different nitrogen concentrations. **(B)** Transcript levels of *carRA, carB,* and *carS* genes at two nitrogen concentrations. The three strains were grown in DG medium for 3 days at 30°C in darkness with 3 g/L^−1^ NaNO_3_ (High N) or with 0.625 g/L^−1^ NaNO_3_ (Low N). Levels of mRNA were determined by RT-qPCR and referred to the levels in the wild type grown in High N, taken as 1. The Petri dishes were incubated for 24 h more before extraction of carotenoids.

The incubation of the three strains in the low nitrogen medium (Low N) resulted in a higher carotenoid content than in excess of nitrogen (High N) (Figure 3A). This correlated with an apparent increase in the mRNA levels of the *carRA* and *carB* genes in the wild type and the complemented strain, but not in those of the *carS* mutant, which exhibited the opposite pattern (Figure 3B). These results suggest that transcriptional activation of the *carRA* and *carB* genes by nitrogen shortage is at least partially dependent on the function of the CarS protein. However, regardless of the amount of nitrogen in the medium, only minor changes were appreciated in the *carS* mRNA levels.

### Effect of heat shock on carotenogenesis

**FIGURE 4:**
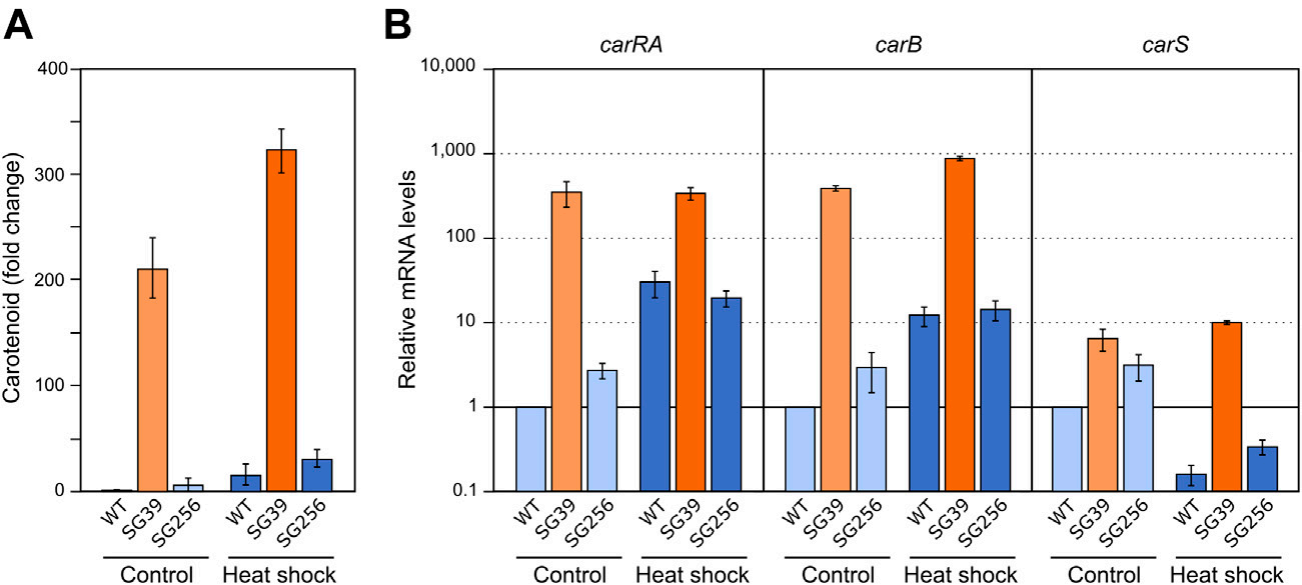
Effect of heat shock on carotenoid production and expression of *car* genes. **(A)** Relative carotenoid production in the wild type (WT), *carS* mutant (SG39), and the complemented mutant (SG256) measured 1 day after 1 hour of heat shock. **(B)** Expression of *carRA, carB* and *carS* genes after 1-h heat shock. The three strains were grown in DG medium for 3 days at 30°C in darkness and incubated for 1 hour at 42°C. RNA was extracted from samples taken after the heat shock and carotenoids from samples incubated 24 h at 30°C after the heat shock. Controls for RNA were incubated 1 hour at 30°C. Controls for carotenoids were incubated 25 hours at 30°C. Levels of mRNA were determined by RT-qPCR and referred to the levels in the wild type grown without heat shock, taken as 1.

In *F. fujikuroi*, *carB* and *carO* genes have been reported to increase their transcript levels in the dark in response to a heat shock of 42°C (Prado et al., 2004; Estrada and Avalos, 2009). However, the expression of the rest of the genes of the cluster (e.g., *carRA*) or *carS* gene, have not been investigated. To carry out this study, the three strains used in the former section were cultivated under the same conditions described for the regulation by light and nitrogen. In this case, after transfer to the Petri dishes, the cultures were incubated for 1 hour at 42°C in the dark. The data showed a result similar to the effect caused by nitrogen starvation (Figure 3A), that is, a detectable increase in the carotenoid content in the three strains after the heat-shock (Figure 4A).

In relation to the effect on gene expression, qRT-PCR analyses revealed an increase in *carRA* and *carB* mRNA levels in the wild type and complemented strains after heat shock (Figure 4B), which could explain the increased carotenoid content. However, this increase by heat shock did not occur in the *carS* mutant, indicating the mediation of CarS in this response. An opposite expression pattern was observed for the *carS* gene, with lower levels after the heat shock. However, the heat shock had hardly any effect on the transcript content of the three genes in the *carS* mutant, while it noticeably increased the amounts of carotenoids also in this strain. In addition, in this experiment it was particularly patent an increase in *carS* mRNA in the *carS* mutant, an effect already described (Ruger-Herreros et al., 2019), that can be attributed to a negative control of CarS protein on transcription of its own gene.

### Effect of H_2_O_2_ on *F. fujikuroi* growth

**FIGURE 5:**
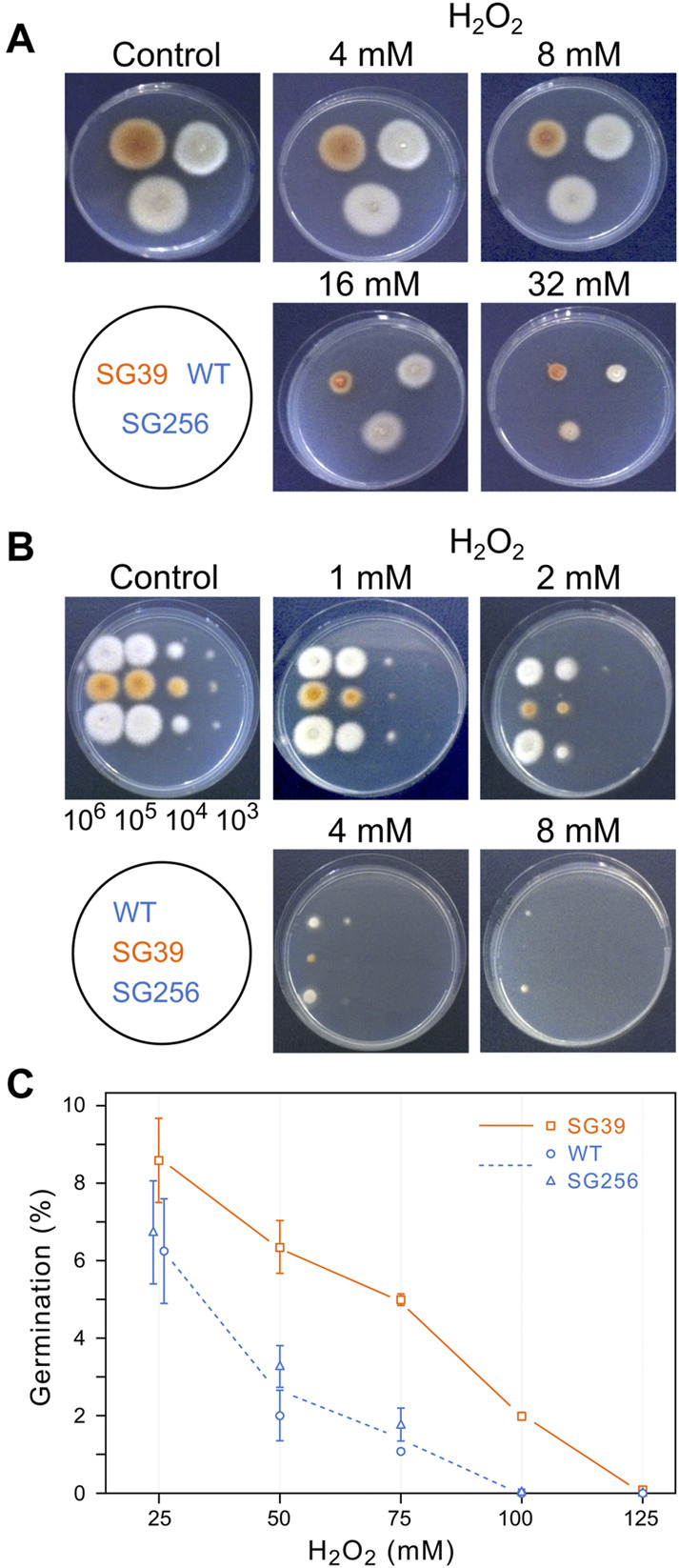
Effect of hydrogen peroxide on growth of the wild type (WT), *carS* mutant (SG39), and the complemented mutant (SG256). **(A)** Growth of surface colonies on media with different H_2_O_2_ concentrations. Strains were grown for 4 days in DG agar medium and then 1-mm^2^ pieces of mycelia were transferred to DG agar with the indicated H_2_O_2_ concentrations. Pictures were taken after 3 days of cultivation at 30°C in darkness. **(B)** Growth from drops containing serial dilutions of spores of the three strains on DG agar with different H_2_O_2_ concentrations, incubated for 3 days at 30°C in the dark. **(C)** Germination of spores exposed to hydrogen peroxide. Spores were incubated with different concentrations of H_2_O_2_ for 30 min in agitation in darkness. Then the spores were washed twice and spread on DG plates to count number of colonies.

The effect of oxidative stress was studied using oxygen peroxide as oxidative agent. As preliminary information, the sensitivity of wild type *F. fujikuroi* to this reactive oxygen species was determined under the culture conditions described above. H_2_O_2_ was added to the Petri dishes to different final concentrations and growth was determined after additional 24 h. Up to 8 mM H_2_O_2_, the fungal biomass was like that of the control, but at 16 and 32 mM there was a visible drop (data not shown). The effect of hydrogen peroxide was also analyzed on agar cultures, by exposing the strains to H_2_O_2_ throughout all the incubation time. Transplants of mycelium from each strain were transferred onto Petri dishes with different H_2_O_2_ concentrations and incubated for 3 days at 30°C in the dark. According to the diameter of the colonies, the *carS* mutant showed greater sensitivity to hydrogen peroxide than the wild type and the complemented strain, the difference being especially pronounced at a concentration of 16 mM, while at 32 mM the three strains were similarly affected (Figure 5A).

Spore germination is a particularly sensitive stage in the life cycle of fungi. To see the effect of oxidative stress on germination, droplets with decreasing spore numbers, from 10^6^ to 10^3^, were seeded on Petri dishes with minimal medium with different H_2_O_2_ concentrations and incubated in the dark for 3 days at 30°C (Figure 5B). The results showed a greater sensitivity of the spores than that exhibited by mycelium in the former experiment. Thus, while in the presence of 8 mM H_2_O_2_ there was no difference in mycelial growth of the strains compared to the control Petri dish without hydrogen peroxide, development from spores was almost non-existent at this concentration. Regarding the comparison between the three strains, the *carS* mutant also showed more sensitivity in these experimental conditions, with the difference especially evident at a concentration of 2 mM H_2_O_2_.

These results indicate that the mycelium of the SG39 *carS* mutant is less tolerant to hydrogen peroxide than the wild-type strain. This difference is caused by the mutation in the *carS* gene, as reveals the recovery of the tolerance in the complemented strain. To confirm this observation, the effect of hydrogen peroxide on the germination capacity of the spores was checked by incubating them for 30 min with different concentrations of H_2_O_2_. The number of colonies growing on DGagar medium indicated the ability of the spores to survive to this stress (Figure 5C). A higher survival rate was found in spores of the *carS* mutant at any of the tested H_2_O_2_ concentrations than for the spores of the wild type or the complemented strains, while in the spore serial dilution experiments (Figure 5B) the growth of SG39 mutant was reduced in comparison to the growth of the *carS*+ strains. From both experiments, we conclude that the spores of the *carS* mutant are more tolerant to a temporary exposure to hydrogen peroxide than those of the wild type, but they are more sensitive to development in the presence of the oxidizing agent.

### Effect of H_2_O_2_ on carotenogenesis

**FIGURE 6:**
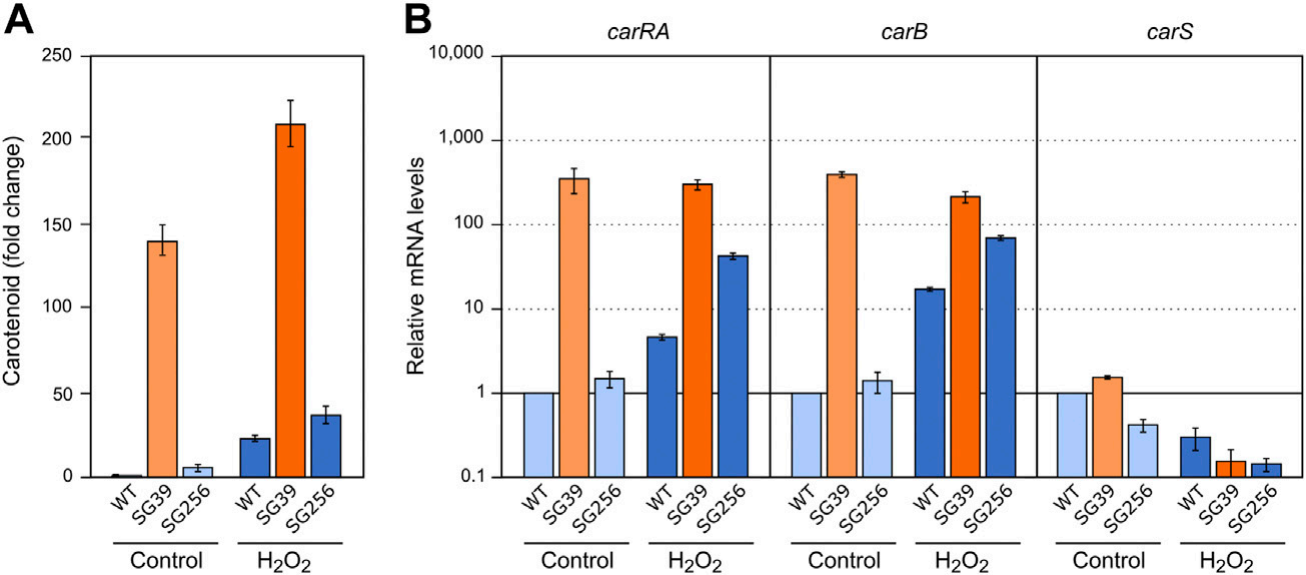
Effect of oxidative stress and *carS* gene mutation on carotenoid production and expression of *car* genes. **(A)** Relative carotenoid accumulation after hydrogen peroxide treatment for 24 h in the wild type (WT), *carS* mutant (SG39), and the complemented mutant (SG256). **(B)** Expression levels of *carRA, carB* and *carS* genes after 1 hour incubation with H_2_O_2_. Control: samples without treatment. Culture conditions are as those for Figure 2. Levels of mRNA were determined by RT-qPCR and referred to the levels in the wild type grown without hydrogen peroxide, taken as 1.

To check the effect of hydrogen peroxide on carotenogenesis, H_2_O_2_ was added to a final concentration of 16 mM to the Petri dishes with the 3-days mycelia of the *carS* mutant and the two control strains and checked for gene expression and carotenoid production. As formerly found for the effects of nitrogen starvation and heat shock, the three strains accumulated more carotenoids after 24 h in the presence of H_2_O_2_ (Figure 6A). In the wild type and the complemented strains, this effect may be attributed to enhanced expression of the *car* genes, as indicates the higher mRNA levels for the genes *carRA* and *carB* found 1 hour after the addition of hydrogen peroxide, whereas no significant changes were observed in these levels in the *carS* mutant (Figure 6B). Interestingly, mRNA amounts of the *carS* gene decreased after H_2_O_2_ exposure in the three strains investigated.

### Effect of light and stress on alternative splicing of the *carS* mRNA intron

**FIGURE 7:**
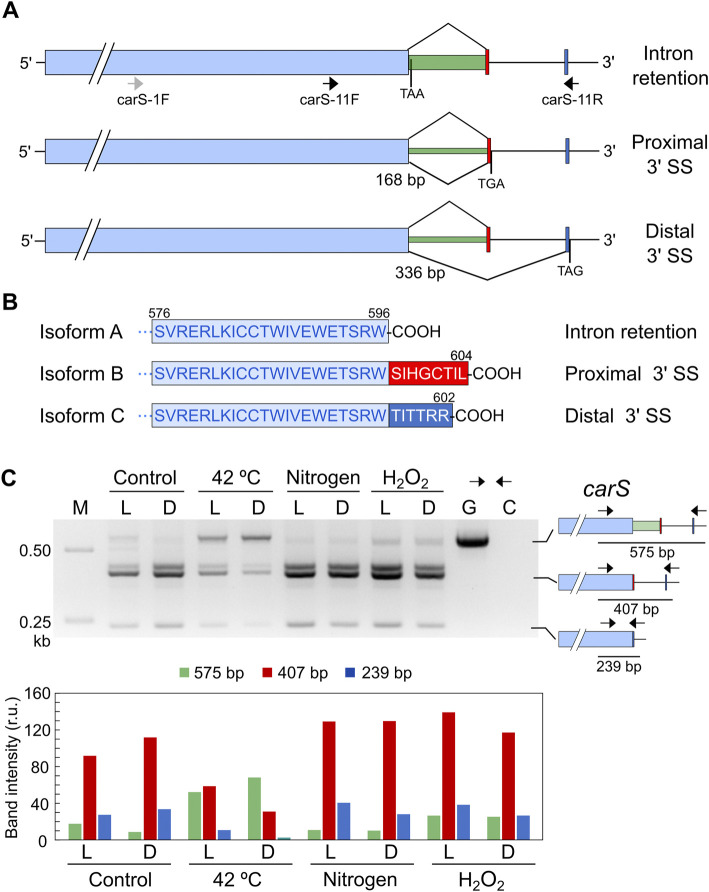
Effect of stressing conditions on splicing of *carS* mRNA. **(A)** Isoforms of *carS* mRNA according to RNA-seq reads. Red and blue bars indicate the mutually exclusive exons fused to the large pale blue exon depending on the splicing event. Arrows indicate primers used in the PCR assays. Stop codons resulting from each mRNA are indicated. Above: intron retention. Middle: splicing of a 168-bp intron. Below: splicing of a 336-bp intron. Only the relevant *carS* segment is displayed. **(B)** Three predicted isoforms of the CarS carboxy end according to the three intron maturation alternatives: intron retention (isoform A), 168-bp intron splicing (isoform B), and 336-bp intron splicing (isoform C). **(C)** PCR test of alternative splicing of *carS* gene in response to stress. Samples of 25 ml of 3-day-old cultures incubated at 30°C in the dark were transferred to Petri dishes and kept under the indicated stress for 1 h in the dark (D) or under illumination (L). Nitrogen starved mycelia (labelled as Nitrogen) were grown for 3 days in medium with 0.625 g/L^−1^ NaNO_3_ before the transfer to Petri dishes. Oxidative stress was generated incubating mycelia with 16 mM H_2_O_2_ for 1 h in the Petri dishes. M, Size markers; G, Genomic DNA; C, Control without cDNA. Molecular interpretation of the PCR products in the three mRNA alternatives is depicted on the schemes on the right. Densitometry analysis of the indicated bands is shown below. Intensity of each band is represented with a different color: green, 575-bp band (intron retention); red, 407-bp band (168-bp spliced intron); blue, 239-bp band (336-bp spliced intron).

Analysis of a global RNA-seq of *F. fujikuroi* (Ruger-Herreros et al., 2019) led us to identify two alternative splicing forms of an intron located in the 3′ region of the *carS* gene (Supplementary Figure S2), which were not included in the *F. fujikuroi* genome annotation available in Fungi DB. Depending on the splicing pattern, two alternative small exons were mutually exclusive. Moreover, a detailed view of the RNA-seq data revealed that the intron was not always removed. Therefore, depending on the splicing event, three different mRNAs may be detected that correspond to intron retention or removal of a short or a large intron (Figure 7A). The removal of the 168-bp intron occurs when a proximal 3′ splicing site (SS) is used while the 336-bp intron is spliced when the distal 3′ SS is chosen. This predictably would lead to three CarS protein isoforms with changes in the carboxy-end sequence (Figure 7B).

To check a possible regulation of CarS activity at mRNA splicing level, we analyzed the effect of light and the different stressing conditions on the generation of the different alternative *carS* transcripts. The occurrence of the three alternative mature mRNAs identified in the RNA-seq study was checked by PCR using cDNA obtained from samples of RNA of the wild type and appropriate primers. In the dark, the three alternatives were identified, with a predominance of the removal of the small intron (Figure 7C), corresponding to the use of the proximal 3’ SS. An additional band was detected above the 407-bp band (Figure 7C) that was not observed with a different pair of primers. This amplification, which does not affect the conclusions, was probably due to non-specific binding of one of the two primers at a point close to the genuine one.

**FIGURE 8:**
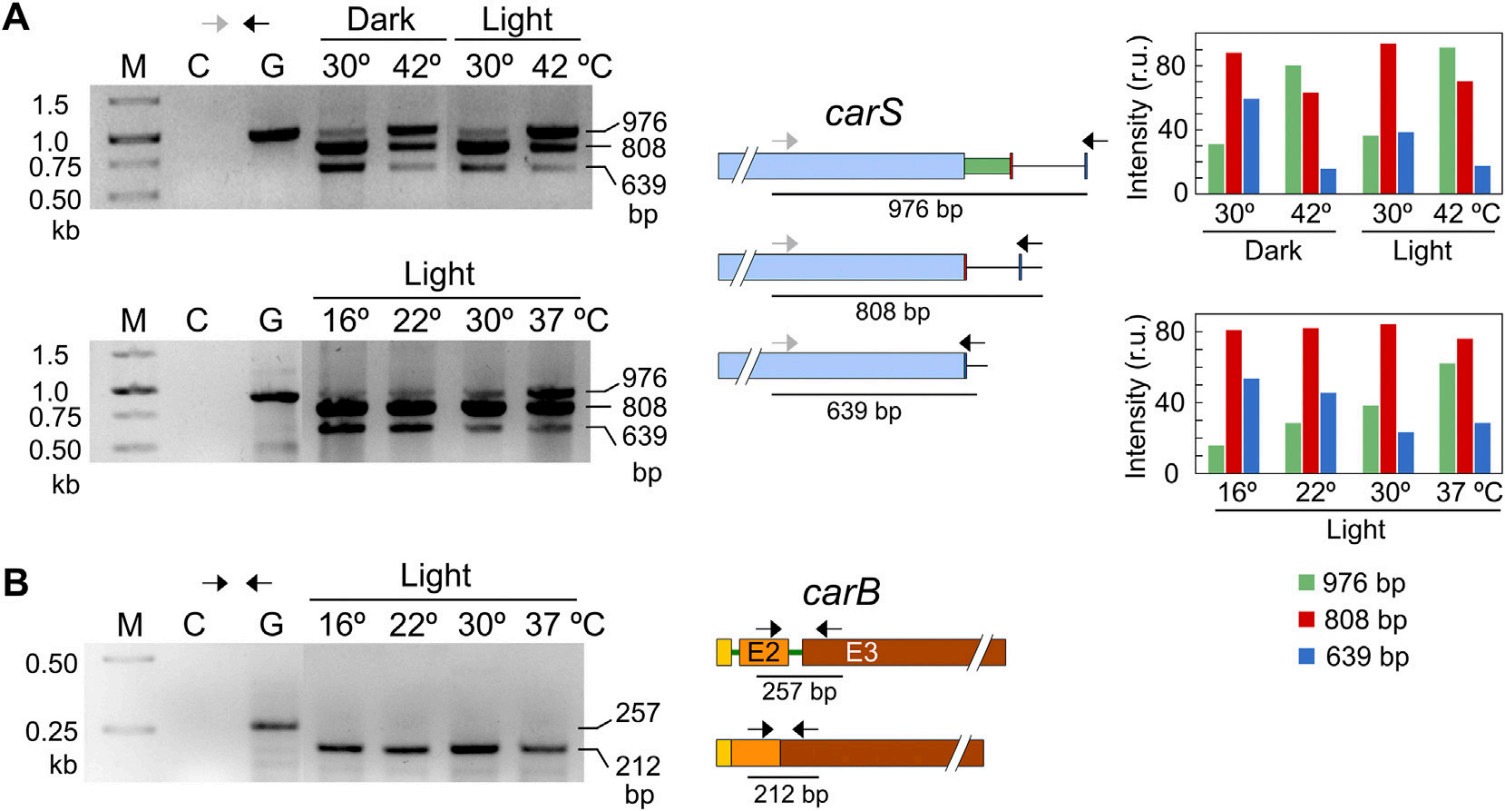
Effect of temperature on mRNA splicing. **(A)** PCR tests of the effect of temperature on splicing in *carS* mRNA in response to temperature. Cultures transferred to Petri dishes were incubated for 1 hour at 30°C in the dark for adaptation and incubated afterwards for an additional hour at the indicated temperature. The correspondence of the bands with the alternative splicing events is shown on the right scheme. In this case, the higher distance between the primers results in larger PCR products compared to Figure 7. Densitometry analysis of the indicated bands is shown on the right. Intensity of each band is represented with a different color: green, 976-bp band (intron retention); red, 808-bp band (168-bp spliced intron); blue, 639-bp band (336-bp spliced intron). **(B)** Effect of temperature on the splicing of the second intron of *carB* gene, which does not exhibit alternative splicing according to RNA-seq data. In the three electrophoresis pictures: M, Size markers; G, Genomic DNA; C, Control without cDNA.

The pattern of alternative splicing forms was very similar after 1-h illumination, under nitrogen starvation or exposure to H_2_O_2,_ (Figure 7C). However, after the heat-shock treatment an increased amount of unspliced mRNA was detected, which corresponded to the largest PCR product, suggesting that this stress provoked a higher proportion of intron retention. This result was confirmed using a primer set that gives amplicons of longer length and allowed a better visualization of the smallest band amplified after the splicing of the larger intron, of 336 bp (Figure 8A). The retained intron contains a premature stop codon at the beginning of its sequence which would produce a truncated CarS protein if the transcript were translated.

Our strains are unable to grow at 42°C, but exhibit a residual growth at 37°C. To check if temperature may play a key role in *carS* regulation through intron retention under physiological conditions, experiments were done with variable temperatures, from 16 to 37°C (Figure 8A). The results showed no relevant differences in the splicing pattern at 16, 22 and 30°C, but revealed a significant increase of intron retention at 37°C (band of 976 bp), as observed at 42°C, that correlated with a decrease of the 3’ alternative splicing (band of 639 bp). The more frequent intron retention cannot be attributed to a defective functioning of the splicing machinery at 37°C, since the second intron of the *carB* gene was efficiently removed at any of the employed temperatures, from 16 to 37°C (Figure 8B). This intron was chosen as a control because it was always removed according to the RNA-seq data (Supplementary Figure S2). Therefore, we conclude that intron retention in the *carS* gene at high temperature, as 37°C, is a regulatory event that could be related to the enhanced carotenoid production found under these conditions.

## Discussion

In this work we have analyzed the effect of different types of stress on carotenogenesis in *F. fujikuroi* in comparison to the effect of light, and the possible role that the expression of the regulatory *carS* gene may play in these responses. To facilitate the application of the different types of stress under equivalent experimental conditions, assays were carried out in static submerged cultures in Petri dishes. Photoinduction of carotenoid synthesis in these cultures was not as high as the one obtained under continuous illumination in agar cultures (Marente et al., 2020a). However, the induction of the structural *carRA* and *carB* genes at the mRNA level was remarkably strong, and an appreciable photoinduction of the *carS* gene was observed, which indicates regulatory conditions comparable to those used in our previous works.

In the experiments described here, the three types of stress investigated, nitrogen starvation, heat shock, and oxidative stress, produced increases in the synthesis of carotenoids comparable to that produced by light. The enhanced biosynthetic activity of the pathway after the exposures to stress may be explained by the higher mRNA levels of the *carRA* and *carB* genes observed in our experiments, but these increases were not as high as the drastic mRNA increment for the same genes after 1 hour illumination. The *carS* mutant has mRNA levels of the *carRA* and *carB* genes in the dark like those of the wild type and complementing strains after illumination, but in that case, it was reflected in a much higher content of carotenoids. A probable explanation for this difference is that while the induction of *carRA* and *carB* mRNA levels after the light pulse is transitory in the wild type, as expected from the existence of a photoadaptation mechanism, the higher mRNA content in *carS* mutants is predictably permanent. Consequently, *carS* mutants gradually accumulate more carotenoids as their culture time increases (Avalos and Cerdá-Olmedo, 1987).

Although adaptation is a well-investigated phenomenon in the regulation by light, and also occurs in the regulation by nitrogen starvation (Rodríguez-Ortiz et al., 2009), it is unknown whether the increase in mRNA of the *carRA* and *carB* genes produced by oxidative or heat stress are also transient. The fact that these levels of mRNA are much lower than those produced by 1 hour illumination while the carotenoid response is similar suggests that the mRNA content is maintained for a longer time. Response kinetics assays should be carried out to clarify this point, especially considering that it is not clear that mRNA levels reach their maximum after 1 hour of exposure to heat shock or H_2_O_2_. The inducing effect of hydrogen peroxide was investigated long ago in *F. aquaeductuum* (Bindl et al., 1970). In this species, the kinetics of carotenoid accumulation in response to hydrogen peroxide exposure was somewhat slower than the kinetics of accumulation in response to a 10-min light pulse, and the amount of carotenoids was about six-fold lower. This lesser response was so even considering that, unlike light, H_2_O_2_ remained in the culture for the entire duration of the experiment after its addition. The relationship of the CarS protein with the inductions produced by light and hydrogen peroxide seems to be different, since light produces an increase in *carS* transcript level, while it decreases in the presence of hydrogen peroxide. This result suggests that the induction of carotenogenesis by hydrogen peroxide in *F. fujikuroi* is carried out through a reduction in the expression of the *carS* gene (Figure 1).

The induction of carotenogenesis by heat shock in the wild type could be also explained by a reduction in CarS activity, e.g., high temperature may affect *carS* mRNA degradation or CarS protein stability. However, we found a novel regulatory feature of *carS* that could explain the effect of high temperature on carotenogenesis. We identified three isoforms of *carS* mRNA, due to alternative splicing of its intron, whose relative proportions change with temperature and heat shock. Heat shock inhibits splicing to different extents in many organisms, including fungi (Georg et al., 2009). The heat shock response is mediated by heat shock factors (HSF) which at the same time are also regulated by alternative splicing. Among the known factors that regulate splicing are the serine/arginine-rich (SR) proteins and the heterogeneous nuclear ribonucleoproteins (hnRNPs), which in plants play an important role in the regulation of splicing in response to abiotic stress (Punzo et al., 2020).

The other conditions investigated, light, nitrogen starvation, and oxidative stress, did not alter the *carS* intron splicing pattern, indicating a specific mechanism for heat stress. Alternative mRNA splicing is a widespread phenomenon in fungi (Muzafar et al., 2021). In *Fusarium graminearum*, RNA-seq analysis found different forms of splicing variability in 231 genes, which included exon skipping, intron retention, or alternative 5′ or 3′ splicing sites (Zhao et al., 2013). In our case, we found the occurrence of intron retention and the use of two alternative exons depending on the 3’ splicing site, resulting in three predicted CarS variants that differ by only a few amino acids at their carboxyl terminus. The three CarS putative isoforms conserve the major protein domains, and therefore could be functional variants, but they could differ in their activity, their regulation, or their subcellular localization. Moreover, some mRNA isoforms could be unproductive (García-Moreno and Romao, 2020). In the case of retention of the *carS* intron, a premature stop codon is now present in its reading frame, meaning that it is most likely not translated, consequently causing a reduction of the CarS repressor. This could explain the increase in carotenoid synthesis after heat shock. Non-sense mediated decay, in which a premature termination codon provokes mRNA degradation, has been described in plants (John et al., 2021) and fungi (Gao et al., 2022). Furthermore, pre-mRNAs with retained introns may remain in the nucleus and thus not be translated (Shalgi et al., 2014). Targeted mutation of the conserved 5′ GT sequence of the intron would be a very informative approach to verify the functionality of the unspliced *carS* mRNA.

Under nitrogen starvation, the increased expression of *carRA* and *carB* genes cannot be attributed to a decrease of *carS* mRNA level, suggesting that carotenogenesis activation occurs in this case through a molecular mechanism different to those due to heat or oxidative stress. Nitrogen regulation could be post-transcriptional, *e.g.*, through *carS* mRNA translation or CarS protein activity, mechanism that could also be involved in the activation of carotenogenesis by light. Interestingly, former data revealed that the *carS* mutants contain higher levels of *carS* mRNA (Ruger-Herreros et al., 2019), indicating a down-regulating role of CarS on its own transcription. A possible inactivation of the CarS protein may explain the tendency for higher *carS* mRNA levels under light. Phenotypic analyses of *carS* mutants revealed effects in other nitrogen-regulated pathways, as those for the syntheses of gibberellins and bikaverin (Rodríguez-Ortiz et al., 2012), supporting the involvement of the CarS protein in nitrogen regulatory mechanisms.

The activation of carotenogenesis by the different investigated stressors may have a common functional cause related to the defense against oxidative stress. In the case of heat stress and nitrogen shortage, both signals may be associated with impending senescence, which in turn will be associated with oxidative stress due to deterioration of mitochondrial functions (Li et al., 2014). In turn, nitrogen scarcity can be interpreted as a signal of imminent growth arrest and subsequent senescence, and a greater accumulation of carotenoids can prevent the increase in the damaging effects of ROS. This biological sense for activation of carotenogenesis is more evident in the case of hydrogen peroxide. These pigments, especially xanthophylls, have been described as antioxidant agents in fungi (Avalos and Limón, 2015). In *Neurospora crassa*, the synthesis of carotenoids in response to light is greater in the absence of superoxide dismutase activity (Yoshida and Hasunuma, 2004). Recent data showed that NX has a very high antioxidant activity (Parra-Rivero et al., 2020), which agrees with the results of the study of *N. crassa*, and with the induction of carotenoid synthesis provoked by hydrogen peroxide in *F. aquaeductuum* (Bindl et al., 1970). Due to their high concentration of carotenoids, the *carS* mutants could be useful tools to study the possible role of these pigments in oxidative stress. Our data indicate a higher resistance to hydrogen peroxide of the *carS* mutants than the wild-type strain at the level of spore germination, but not of colony development. This apparent contradiction could be explained by the regulatory roles of CarS on the expression of several genes with catalase domains (Ruger-Herreros et al., 2019). Therefore, the CarS protein has other functions besides regulating carotenogenesis genes, complicating the interpretation of its phenotype in responses to stress. Moreover, CarS protein controls directly or indirectly many other cellular functions, as indicated by its impact on the global transcriptome.

As a conclusion, regardless of their possible functional connections, our data suggest that light and the three investigated stressors activate carotenogenesis through different molecular mechanisms. In the case of oxidative and heat stress, both negatively affect mRNA levels of *carS* gene (Figure 1). However, light and nitrogen starvation do not act through a reduction in *carS* transcripts. We cannot rule out the participation of other regulatory proteins but, considering the central role played by CarS in the regulation of carotenogenesis, their activations could be explained by post-transcriptional effects on CarS synthesis or activity. More research is still needed on this fascinating protein to understand the complexity of its regulation and functions in this fungal group.

## Data Availability

All datasets presented in this study are included in the article/Supplementary Material.
