# Supplementary material for: Relation between CarS expression and activation of carotenogenesis by stress in Fusarium fujikuroi
Source: Front Bioeng Biotechnol. 2022 Oct 5;10:1000129. doi: 10.3389/fbioe.2022.1000129 (PMC9581392; doi:10.3389/fbioe.2022.1000129)
Supplement: Supplementary file 1 [file Table1.docx]

Supplementary Material

**Relation between CarS Expression and Activation of Carotenogenesis by Stress in *Fusarium fujikuroi***

Macarena Ruger-Herreros, Steffen Nordzieke, Carmen Vega-Álvarez, Javier Avalos, M. Carmen Limón

# Supplementary Table

Supplementary Table S1. Carotenoid production (µg / g dry mass, average and standard deviation) in the data shown in Figures 2, 3, 4 and 6.

|  | **WT** | **SG39** | **SG256** |
| --- | --- | --- | --- |
| **Light** |  |  |  |
| Control | 11.33 ± 1.35 | 351.01 ± 16.33 | 30.02 ± 6.57 |
| 1h light | 22.18 ± 5.79 | 532.27 ± 65.11 | 48.18 ± 3.43 |
| **Nitrogen** **starvation** |  |  |  |
| High N (3 g L^-1^ NaNO_3_) | 4.90 ± 3.70 | 145.00 ± 23.70 | 13.00 ± 4.81 |
| Low N (0.63 g L^-1^ NaNO_3_) | 26.00 ± 5.56 | 216.00 ± 26.30 | 37.00 ± 9.26 |
| **Heat shock** |  |  |  |
| 30ºC | 2.67 ± 0.00 | 567.21 ± 74.44 | 19.30 ± 15.93 |
| 42ºC | 45.76 ± 26.30 | 863.8 ± 52.59 | 85.98 ± 22.22 |
| **Oxidative stress** |  |  |  |
| Control | 2.65 ± 0.00 | 371.21 ± 23.70 | 14.44 ± 4.44 |
| 16 mM H_2_O_2_ | 60.78 ± 4.81 | 553.50 ± 38.15 | 96.98 ± 14.07 |
