# Supplementary material for: Relation between CarS expression and activation of carotenogenesis by stress in Fusarium fujikuroi
Source: Front Bioeng Biotechnol. 2022 Oct 5;10:1000129. doi: 10.3389/fbioe.2022.1000129 (PMC9581392; doi:10.3389/fbioe.2022.1000129)
Supplement: Supplementary file 2 [file Image2.pdf]

Supplementary Material

Relation between CarS Expression and Activation of Carotenogenesis by Stress in *Fusarium fujikuroi*

Macarena Ruger-Herreros, Steffen Nordzieke, Carmen Vega-Álvarez, Javier Avalos J,

M. Carmen Limón

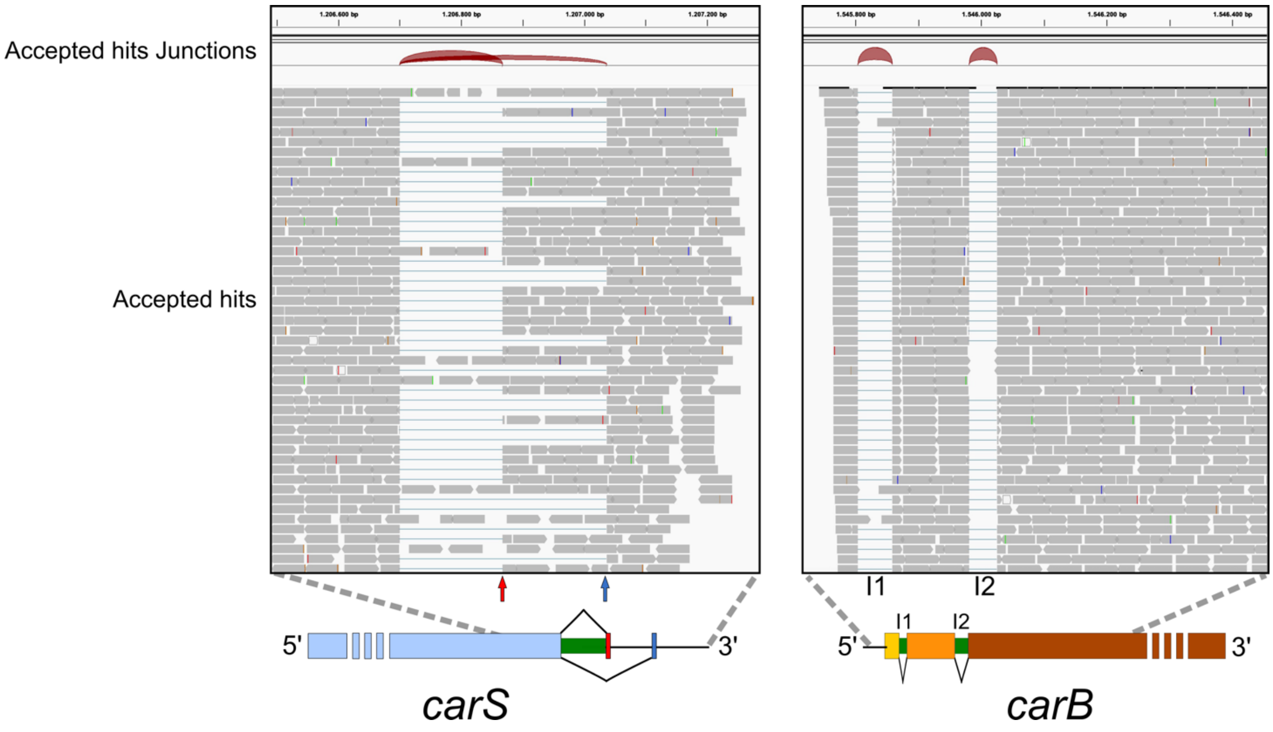

Supplementary Figure S2. Transcript readings (accepted hits) according to RNA-seq data in the genomic sequences corresponding to the 5' region of gene *carS* and 3' region of gene *carB* shown below. Accepted hits junctions indicate exon connections confirmed by continuous readings. Red and blue arrows indicate the alternative 3' splicing sites in the *carS* intron. Intron I2 in *carB* was chosen as splicing control in Figure 7. The readings were represented with IGV program (Robinson et al., 2011, *Nat. Biotech.* 29, 24–26) (https://software.broadinstitute.org/software/igv/).
