# Supplementary material for: Relation between CarS expression and activation of carotenogenesis by stress in Fusarium fujikuroi
Source: Front Bioeng Biotechnol. 2022 Oct 5;10:1000129. doi: 10.3389/fbioe.2022.1000129 (PMC9581392; doi:10.3389/fbioe.2022.1000129)
Supplement: Supplementary file 3 [file Image1.pdf]

Supplementary Material

Relation between CarS Expression and Activation of Carotenogenesis by Stress in *Fusarium fujikuroi*

Macarena Ruger-Herreros, Steffen Nordzieke, Carmen Vega-Álvarez, Javier Avalos J,

M. Carmen Limón

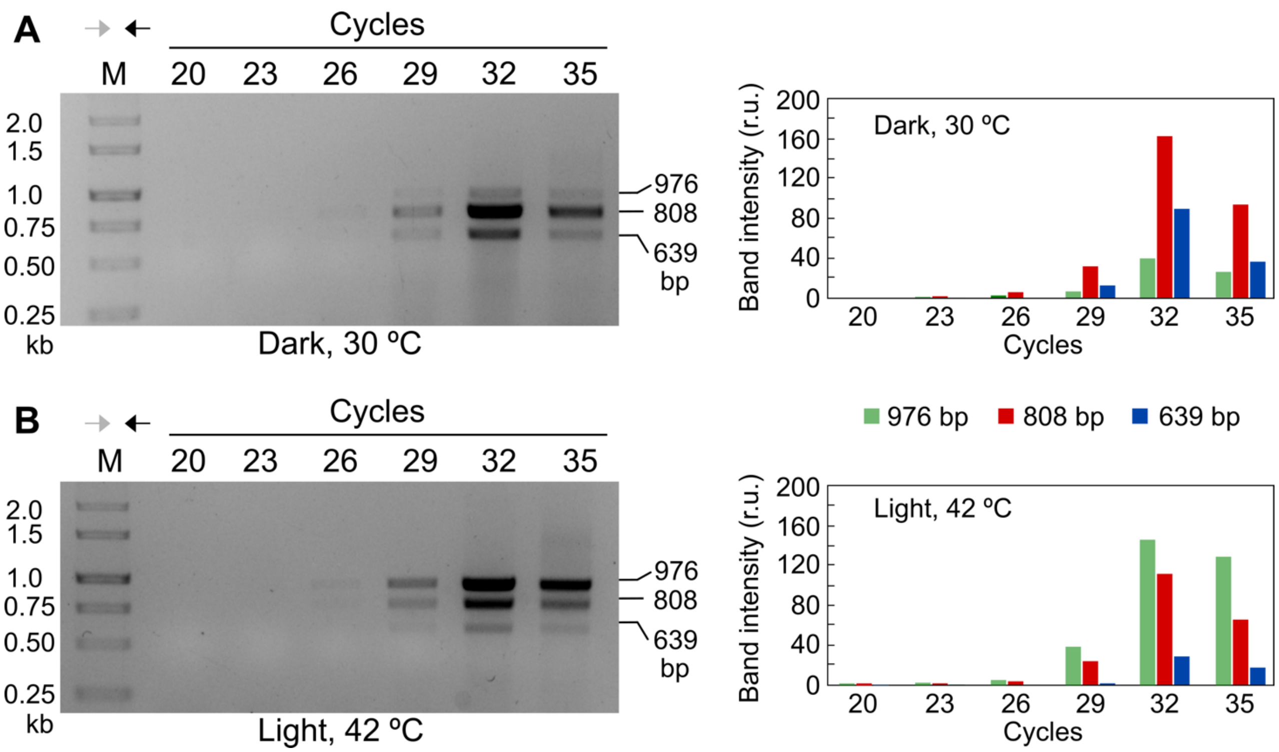

Supplementary Figure S1. Effect of cycle number on the amplification of *carS* mRNA segments. PCR amplifications were carried out using as templates 50 ng of cDNA of two different samples treated with different temperatures. The pictures show electrophoresis of PCR products with the number of amplification cycles indicated above. M, size markers. (**A**) Result obtained from a sample incubated 1 h at 30 ºC in the dark after 1h of adaptation to 30 ºC in the dark. (**B**) Result obtained with a sample incubated 1 h at 42 ºC under illumination after 1h of adaptation to 42 ºC in the dark. Primer set used was S1-carS-F1 and carS-11R. PCR program: 94 ºC 2 min, different number of cycles of 94 ºC 30 s, 58 ºC 1 min, 72 ºC 30 s, and extension at 72 ºC for 10 min. Electrophoreses were run in 1.2 % agarose gels. Densitometry of the bands from each electrophoresis is shown on the accompanying graphs. The amount of product started to be visible from 26 cycles and increased up to 32 cycles. The band of 976 pb corresponds to intron retention and the bands of 808 and 639 bp correspond to different 3' alternative splicing events (see Figure 8). The proportions between the different bands in each graph remained similar up to 35 cycles.
